# Long-Term Maintenance Strategies after Pulmonary Rehabilitation: Perspectives of People with Chronic Respiratory Diseases, Informal Carers, and Healthcare Professionals

**DOI:** 10.3390/healthcare10010119

**Published:** 2022-01-07

**Authors:** Sara Souto-Miranda, Cláudia Dias, Cristina Jácome, Elsa Melo, Alda Marques

**Affiliations:** 1 Lab3R—Respiratory Research and Rehabilitation Laboratory, School of Health Sciences (ESSUA), University of Aveiro, 3810-193 Aveiro, Portugal; sara.souto@ua.pt; 2 iBiMED—Institute of Biomedicine, School of Health Sciences (ESSUA), University of Aveiro, 3810-193 Aveiro, Portugal; elsamelo@ua.pt; 3 Home Care Department, Linde Healthcare, 1200-217 Lisbon, Portugal; claudiasrdias@gmail.com; 4 Center for Health Technology and Services Research (CINTESIS), Faculty of Medicine, University of Porto, 4200-450 Porto, Portugal; cjacome@med.up.pt; 5 Department of Community Medicine, Information and Health Decision Sciences (MEDCIDS), Faculty of Medicine, University of Porto, 4200-450 Porto, Portugal

**Keywords:** chronic respiratory diseases, maintenance strategies, pulmonary rehabilitation, physical activity

## Abstract

Pulmonary rehabilitation (PR) is an effective intervention for people with chronic respiratory diseases (CRD); however, its effects fade after 6–12 months. Community-based strategies might be valuable to sustain PR benefits, but this has been little explored. People with CRD, informal carers, and healthcare professionals (HCPs) were recruited from pulmonology appointments of two local hospitals, two primary care centres, and one community institutional practice and through snowballing technique. Focus groups were conducted using a semi-structured guide. Data were thematically analysed. Twenty-nine people with CRD (24% female, median 69 years), 5 informal carers (100% female, median 69 years), and 16 HCPs (75% female, median 36 years) were included. Three themes were identified: “Maintaining an independent and active lifestyle” which revealed common strategies adopted by people with “intrinsic motivation and professional and peer support” as key elements to maintain benefits, and that “access to information and partnerships with city councils’ physical activities” were necessary future steps to sustain active lifestyles. This study suggests that motivation, and professional and peer support are key elements to maintaining the benefits of PR in people with CRD, and that different physical activity options (independent or group activities) considering peoples’ preferences, should be available through partnerships with the community, namely city councils.

## 1. Introduction

Chronic respiratory diseases (CRD) are leading causes of mortality and years lived with disability worldwide [1]. Pulmonary rehabilitation (PR) is a cost-effective and multidisciplinary intervention known to improve symptoms, exercise capacity, and health-related quality of life of people with CRD, such as chronic obstructive pulmonary disease (COPD), asthma, and interstitial lung disease [2,3,4]. However, unless people keep being physically active, which is highly challenging, these benefits tend to fade after 6 to 12 months of completing a PR programme [5,6].

Engaging people with CRD in maintenance PR programmes for indefinite periods of time might not be the optimal approach, as PR is a highly inaccessible intervention [7] and aims to empower patients to better self-manage their disease and keep a healthy lifestyle [2]. Other strategies to maintain the benefits of PR are therefore needed.

Addressing this relevant issue, studies have been conducted exploring the benefits of maintenance exercise training programmes after PR; however, their effectiveness is still not clear [2]. Community-based maintenance strategies—i.e., physical activity/exercise interventions—might provide a valuable opportunity to sustain PR benefits [6], as they are commonly provided by city councils or local organisations to the general population being accessible and convenient [8,9,10]. 

To successfully implement such strategies, the needs and expectations of people with CRD and informal carers’ need to be considered and meet healthcare professionals’ (HCPs) goals. Nevertheless, their perspectives on long-term maintenance strategies for people with CRD are yet unknown. This study aimed to explore which maintenance strategies should be available for people with CRD in the community and how they should be provided, from the perspectives of people with CRD, informal carers, and HCPs.

## 2. Materials and Methods

### 2.1. Study Design

A qualitative exploratory study with focus groups (group interviews) was conducted. This study is nested in a larger project approved by three independent Ethic Committees (UAI F 83/2019; P517-08/2018; 086892). All participants provided written informed consent.

The study is reported following the Consolidated Criteria for Reporting Qualitative Research (COREQ) [11] and the standards for reporting qualitative research (SRQR) [12].

### 2.2. Participant Selection

Participants from three stakeholder groups were recruited: people with CRD, informal carers, and HCPs. People with CRD and HCPs were recruited face-to-face using purposive sampling [13] from the pulmonology appointments of two local hospitals, two primary care centres, and one community institutional practice. People with CRD and HCPs were first identified by staff of these institutions, not involved in the study, who explained the study briefly. Then, participants who were interested were contacted by one member of the research team (C.D.). 

Informal carers were recruited through people with CRD, using the snowballing technique [13]. People with CRD had to be adults with a clinical diagnosis of a CRD (e.g., COPD, asthma, interstitial lung disease) and had participated at least once in a PR programme. Informal carers were eligible if they were adults, the most significant person for the person with CRD, and if they provided physical or practical (e.g., in activities of daily living, healthcare), social, financial, and/or emotional support to the person with CRD [14]. People with CRD, or their informal carers, were excluded if they had difficulty communicating or understanding, history of abuse of substances, or a diagnosis of a significant psychiatric disorder.

HCPs were eligible if they had been directly involved in the implementation of PR programmes.

The pulmonary rehabilitation programme prior to the focus groups followed the international recommendations and was “a comprehensive intervention based on a thorough patient assessment followed by patient-tailored therapies” [2], that included exercise training (60–70 min, 2 times per week), education and psychosocial support and behaviour change (once every 2 weeks) for 12 weeks. Aerobic training was performed for 20–30 min at 80% of the average speed achieved during the 6-min walk test, and resistance training consisted of 8 exercises of the major upper and lower limb muscle groups, at 60 to 70% of 1-RM. Medical doctors, nurses, physiotherapists, nutritionists, psychologists, and social workers were involved in the programme.

Sample size estimations in thematic analysis should consider the richness and complexity of data for addressing the research question rather than a specific number obtained with a power calculation [15]. For medium-sized studies similar to ours, a sample size gathered in 3 to 6 focus groups or 10–20 interviews has been advised [16]. A total of 74 participants—29 people with CRD, 29 informal carers, and 16 HCPs—were invited to participate in this study.

### 2.3. Data Collection

All participants provided information regarding their age, gender, education level, marital status, and occupation.

For people with CRD, height and weight were measured and data from spirometry were retrieved from medical records. Additionally, smoking status, use of long-term oxygen therapy, non-invasive ventilation, number of acute exacerbations and hospitalisations in previous year, and impact of the disease with COPD assessment test, were also collected.

Informal carers were asked about the kinship with the person with CRD, geographical distance to their house, and duration of care provided.

HCPs also provided their professional background, years of professional experience, and the setting of their workplace.

Focus group interviews were conducted separately with each stakeholder group containing 6 to 12 participants [17] in a room with only the participants and the moderator (C.D. or A.M.). A semi-structured guide with open-ended questions, that was first pilot tested with one focus group of 5 people with CRD, was used to allow an interactive discussion. Two recorders (WS–750 m, Olympus, Tokyo, Japan) were used to ensure audio quality and avoid missing data. Then, audio files were saved to a computer with restricted access only to the researchers and the original audios were deleted from the recorders. To ensure confidentiality, names of the participants were replaced with pseudonyms. After each focus group, the moderator made field notes with the most important messages and ideas for analysis.

### 2.4. Research Team and Reflexivity

C.D. contacted participants, conducted five focus groups, transcribed all data, and analysed the data first. She is a female experienced physiotherapist, with a master’s degree in respiratory physiotherapy and clinical practice in assessing and treating people with CRD in a hospital. 

A.M. conceived the idea, drafted the interview guide, pilot-tested it, conducted two focus groups, reviewed the transcripts, was involved in data analysis and interpretation, and supervised C.D. during her master’s degree. A.M. is a female experienced respiratory researcher in pulmonary rehabilitation and qualitative studies who holds a PhD.

Before the start of the study, there was no relationship established between the moderators and the participants. However, the interviewers presented themselves to the participants who were aware of their motivations for conducting the research. The interviewers had knowledge of the importance of PR and were aware of the challenges to maintain a healthy lifestyle. 

S.S.-M. is a female PhD candidate with a master’s degree in respiratory physiotherapy and experience in assessing and treating people with CRD, as well as in conducting qualitative studies. S.S.-M. analysed the data, interpreted it, and prepared the manuscript.

E.M. reviewed the transcripts, was involved in data analysis and interpretation, and co-supervised C.D. during her master’s degree. E.M. is an experienced female nurse who collaborates with PR, conducts qualitative studies, and holds a PhD.

C.J. is a female researcher that holds a PhD with extensive knowledge in PR and qualitative studies. C.J. analysed the data, interpreted it, and revised the manuscript. 

### 2.5. Data Analysis

Descriptive statistics were computed using SPSS software (v26, IBM, Armonk, NY, USA).

Qualitative data were analysed with inductive thematic analysis in 6 phases: transcription, generating initial codes, searching for themes, reviewing themes, defining and naming themes, and producing the report [18]. The focus groups were transcribed by one researcher (C.D.) and checked for accuracy by two other members of the team (A.M and E.M.).

One author (C.D.) generated the initial codes using organic coding and the codes were then merged and interpreted as themes, when there were common patterns within the data [16]. During the analysis, memos were used to register decisions and other meaningful notes. Themes were discussed with the research team (S.S.-M., C.D., E.M., C.J. and A.M.) until consensus was reached. Member checking (i.e., participants read the results to ensure they were accurately describing their thoughts) was performed with four participants, two people with CRD and two HCPs, to ensure credibility and trustworthiness of findings [19]. No qualitative software was used.

### 2.6. Trustworthiness

Trustworthiness, procedures of credibility, transferability, dependability, and confirmability were ensured (Table 1), as recommended [20].

## 3. Results

All people with CRD and HCPs agreed to participate. Only 5 of 29 informal carers contacted participated, as they did not perceive their input as valuable, or due to time unavailability. A total of 50 people (29 people with CRD, 5 informal carers, and 16 HCPs) were included in the study. Seven focus groups (four with people with CRD, one with informal carers, and two with HCPs) were conducted. Focus groups lasted on average 47 ± 15 (32–70) minutes. People with CRD were mostly male (76%), with COPD (83%), with a median age of 69 years old, and mostly of GOLD group B (48%). Informal carers were all females, with a median age of 69 years old, mostly spouses (80%). HCPs were mostly females (75%), with a median age of 36 years old, working mainly at hospitals (62.5%). Table 2 describes the characteristics of participants.

**Figure 1:**
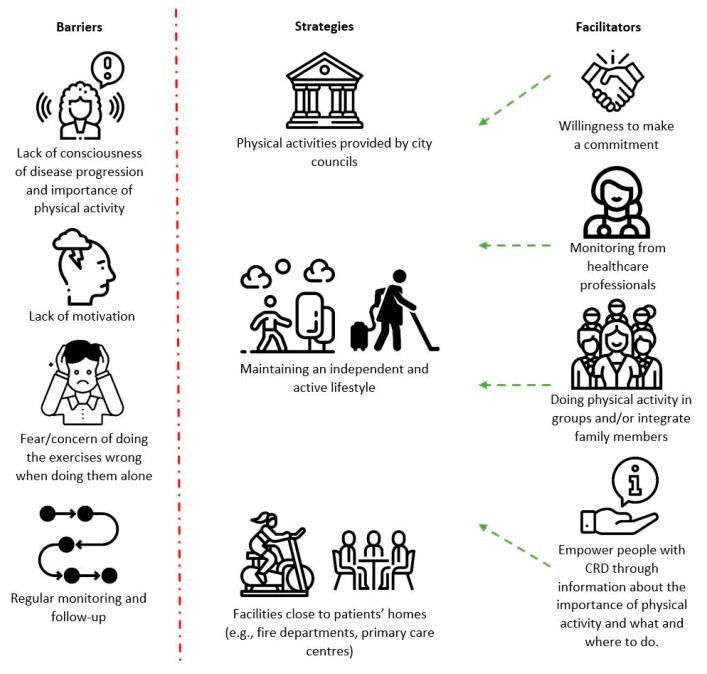
Maintenance strategies, barriers, and facilitators identified by people with chronic respiratory disease (CRD), informal carers, and healthcare professionals. Images freely downloaded from flaticon.com, accessed on 4 January 2022.

Three themes explaining the perspectives of the different stakeholders were identified: “maintaining an independent and active lifestyle”, “intrinsic motivation and professional and peer support”, and “partnerships with city councils’ physical activities”. No differences in perspectives between stakeholder groups or subgroups within each stakeholder group were found. Figure 1 shows the different strategies, barriers, and facilitators to maintain the benefits of PR, identified throughout the themes. The themes are described in detail below.

### 3.1. Maintaining an Independent and Active Lifestyle

Several physical activities such as walking or doing house chores were identified as commonly used strategies adopted by people with CRD to keep the benefits of PR and an active lifestyle on their own. People expressed that activities such as walking, cycling, or vacuum cleaning made them feel physically active, which was crucial to maintaining their well-being. Additionally, they felt that physical activity could be done in several places, such as patients’ homes, community centres, and outdoors—taking advantage of green spaces with clean air. Nonetheless, all stakeholders felt that these activities should consider people’s preferences to help them maintain their motivation and prevent dropouts.

*“(…) I do physical activity at home, like vacuum cleaning, it’s also good for me. I also climb a lot of stairs at home.”*

—J., male, 61 y, COPD

*“Walking… I had no idea that it was so good for him, and he got excited with counting his steps.”*

—A., female, 53 y, informal carer

*“If the patient has a bicycle or likes to walk, we have to give positive reinforcement. We need to work with them while they’re here to try to figure out what they do and what they like.”*

—J., male, 33 y, MD

### 3.2. Intrinsic Motivation and Professional and Peer Support

Participants thought one of the most important barriers to being physically active was the lack of motivation in people with CRD and that this was related with the lack of awareness or consciousness about their condition. People with CRD felt that some of them, when not fully aware of how the disease progresses, might neglect physical activities by thinking they are not needed. They expressed that it was necessary to make a commitment with a professional, so they would feel inhibited from missing the session. Informal carers also revealed that keeping people with CRD engaged in social physical activities or maintenance PR made them feel at ease and better themselves, by seeing their loved ones were feeling happier and capable of doing more tasks at home. All stakeholders felt that giving the power to choose the type of activity to people with CRD and performing group activities would foster their motivation and could help break their typical social isolation. Furthermore, people with CRD showed that they were often scared of having no professional monitoring and that doing the activities by themselves could be detrimental, as they were not confident on how to perform the movements correctly. All stakeholders felt that, to keep people with CRD motivated and feeling safe, the activities should integrate the family and be monitored by a qualified professional. They also expressed that frequent follow-up should be done, to provide feedback to people with CRD on the course of their disease and reaching their goals and make any necessary adjustments.

*“I have equipment at home, I do one day then I am one month without doing it, then I’ll do it again. This does absolutely nothing. Because there’s no motivation, and there’s fear, fear of doing it wrong.”*

—T., female, 69 y, COPD,

*“It’s for their own good. Theirs and ours. Because their well-being is our well-being.”*

—C., female, 69 y, informal carer

*“Maintaining frequent physical activity with supervision, that’s what we saw that works, because without supervision their motivation decreases, and they stop doing physical activity.”*

—A., female, 35 y, Physiotherapist

### 3.3. Access to Information and Partnerships with City Councils’ Physical Activities

Participants felt that to induce behavioural change in people with CRD, they should be empowered through access to information about the types and places of available activities, and through awareness of the importance of being physically active. Participants felt that community PR programmes should also be available, so patients could maintain the benefits of the first programme conducted in specialised institutions. They also thought that creating partnerships with city councils would be of value for both interested parties, as PR services could refer people with CRD to physical activities and city councils could identify people with CRD that should be integrated in PR. Additionally, participants felt that city councils would have the necessary resources to place qualified professionals in monitoring people with CRD, close to people’s homes, such as fire departments, parish councils’ facilities or primary care centres, to conduct different physical activities at reduced costs.

*“(…) but pulling them to where we are, our centre, our region. Because this cost a lot of money, right? The cars don’t move on water, it’s difficult to bear.”*

—F., male, 67 y, interstitial lung disease

*“But I think that primary care centres are the closest to people, so I think that it will have to be in primary care centres.”*

—C., female, 69 y, informal carer

*“The city councils, they have their own health departments now, and they know what institutions they have in the region. It would be good if we could ask them to disseminate our programme and they told us what activities exist and in what institutions. Because usually, every institution that exists in every council is funded by the city council.”*

—A., female, 33 y, Physiotherapist

## 4. Discussion

This study showed the perspectives of people with CRD, informal carers, and HCPs on maintenance strategies following a PR programme.

Our findings revealed that stakeholders thought people with CRD could perform some physical activities without supervision, such as walking or house chores, to keep an independent and active lifestyle. Studies have found that people with CRD often adopt more active lifestyles after PR, through occupational and household activities, walking, or cycling [21]. Nevertheless, adopting and sustaining this behaviour once PR is complete is highly challenging [22,23] and solely recommending that people with CRD be active by themselves as a ‘one size fits all’ approach might not be effective. More personalised interventions are therefore needed.

A recent review found weak evidence for the effectiveness of exercise programmes following PR in people with COPD [8]. Authors have however acknowledged that these programmes did not take into consideration the preferences of patients [8]. Our study shows that stakeholders believe that creating partnerships with existing physical activities of city councils might be a good alternative, as they can take the preferences of people into account, be supervised, and can be performed in groups including family members. However, we did not find studies of such initiatives which need to be explored.

This study suggests motivation and peoples’ preferences to be the key propulsors to engage people in active lifestyles. Doing physical activities with peers under the supervision of an HCP were recognised in our study, but also by others as facilitators to keep people with CRD motivated and adherent to interventions [8,9,10]. Moreover, higher self-determined motivation has been associated with better exercise behaviour [24] and was acknowledged by people with CRD in our study as a determinant for active lifestyles. Hence, the use of techniques to increase motivation, such as motivational interviewing or physical activity coaching, might be necessary to encourage active lifestyles and maximise the long-term response to PR [6,25,26,27]. Therefore, assessing motivation (i.e., motivational interviewing), to personalise these interventions, providing professional support and having different physical activity options (individual and groups) available seems important. Regarding professional support, since most of the physical activities provided by city councils are already delivered by qualified professionals (i.e., sports professionals), providing respiratory-specific training to them might be a good strategy to optimize resources on the long-term whilst ensuring the safety and effectiveness of the interventions.

Currently, physical activities adapted for people with CRD in the community are scarce, and since monitoring was highly valued by participants, their interest of having maintenance PR programmes in the community (e.g., primary care centres) was not surprising. Other studies have also shown that having maintenance PR after a first programme is often a desire of people with CRD [28], and that it is beneficial in terms of exercise capacity of people with COPD [29]. Hence, this approach could be one of the available options for people with CRD. Nonetheless, it might not be the optimal long-term strategy, as this is a highly inaccessible intervention, with several barriers for people with CRD, such as geographical distance and costs, and its ability to improve the physical activity levels is not clear [22]. Other solutions need to be pursued.

Finally, focus groups were a suitable methodology to gather stakeholders’ views as this topic was unexplored [16]. An in-depth understanding can now be obtained by conducting individual interviews in future studies.

This study has some limitations. Although efforts were made to have a balanced sample, the recruitment of informal carers was insufficient, and their views might not be representative of this stakeholder group. Difficulties in recruiting loved ones have been previously acknowledged in other studies [30]. Furthermore, given that only 5 of the 29 patients had ILD, future studies could explore the views of this group separately and compare them with our results. Therefore, future studies should try to engage informal carers and seek their perspectives on the optimal way to deliver maintenance interventions. This study was also conducted with non-English speakers. Although translations were made by proficient English speakers, some thoughts could have been missed in translation. Our purpose was to explore stakeholders’ perspectives in a qualitative study to complement the quantitative data available in the literature. Nevertheless, future mix-method studies might provide additional valuable information to further enhance our knowledge about the maintenance strategies that should be available for people with CRD in the community.

## 5. Conclusions

This study suggests that motivation, and peer and professional support are key elements to maintaining the benefits of PR in people with CRD, and that different options considering people’s preferences, such as independent activities (walking, household activities) or group-based activities provided by city councils, should be available.

## Figures and Tables

**Table 1:** Procedures to ensure trustworthiness of analysis and results.

Criteria	Description of the Procedures Performed
Credibility	Ensured by (i) team meetings among all members of the research team were conducted to compare the analysis and agree on final categories and themes; (ii) participant triangulation, i.e., participants were recruited from different settings (hospitals and community) to obtain multiple perspectives with a common purpose; (iii) triangulation of methods collection, i.e., focus groups interviews were conducted, and researcher field notes were kept; (iv) participant validation, i.e., findings were presented to four participants—2 people with chronic respiratory disease and 2 healthcare professionals—for confirmation. Findings were confirmed and additional comments were not provided; (v) researcher reflexivity, i.e., describing the rationale of the study and discussing it as well as the analyses with the other researchers.
Transferability	Ensured by describing the study in detail; sampling strategies, characteristics of researchers (their role and background), participants and sites/contexts of data acquisition, as well as all procedures of the analysis.
Dependability	Ensured by having an external researcher (ACG) assessing the research protocol and focus groups interview guide who also checked the coded descriptions, themes and sub-themes, participant quotations, and quotation identification.
Confirmability	Ensured by the investigator, participant and data collection triangulation and researcher reflexivity was encouraged, conducted in a written format, and discussed among all team members and with the external researcher.

**Table 2:** Characteristics of the participants included (*n* = 50).

Characteristic	People with CRD (*n* = 29)	People with COPD (*n* = 24)	People with ILD (*n* = 5)	Informal Carers (*n* = 5)	HCPs (*n* = 16)
Age, years median (min–max)	69 (45–79)	69 (50–79)	66 (45–78)	69 (53–72)	36 (27–61)
BMI, kg/m^2^	26.3 ± 5.1	25.9 ± 5.5	27.0 ± 1.9	-	-
Sex, *n* (%)					
Female	7 (24.1)	6 (25.0)	1 (20.0)	5 (100)	12 (75.0)
Male	22 (75.9)	18 (75.0)	4 (80.0)	0 (0)	4 (25.0)
FEV_1_, % predicted	57.3 ± 22.3	51.7 ± 19.3	83.2 ± 16.9	-	-
FVC, % predicted	80.2 ± 21.0	81.0 ± 21.8	76.5 ± 18.3	-	-
GOLD group, *n* (%)					
A	-	6 (20.7)	-	-	-
B	-	14 (48.3)	-	-	-
C	-	4 (13.8)	-	-	-
D	-	5 (17.2)	-	-	-
Smokers, *n* (%)					
Current	2 (6.9)	2 (8.3)	0 (0.0)	-	-
Former	16 (55.2)	13 (54.2)	3 (60.0)	-	-
Never	11 (37.9)	9 (37.5)	2 (40.0)	-	-
LTOT, *n* (%)	2 (6.9)	1 (4.2)	1 (20.0)	-	-
NIV, *n* (%)	5 (17.2)	5 (20.8)	0 (0.0)	-	-
AECOPD, number previous year, median (IQR)	0 (0–2)	0 (0–2)	0 (0–0)	-	-
Hospitalisations, number previous year, median (IQR)	1 (1–1)	1.0 (1–1)	1 (1–1)	-	-
CAT, score	13.8 ± 7.7	14.4 ± 7.2	11.0 ± 9.4	-	-
Occupation, *n* (%)					
Retired	25 (86.2)	20 (803.3)	5 (100)	1 (20.0)	0 (0)
Housework	2 (6.9)	2 (8.3)	0 (0)	2 (40.0)	0 (0)
Paid work	2 (6.9)	2 (8.3)	0 (0)	2 (40.0)	16 (100)
Profession, *n* (%)					
Medical doctor	-			-	5 (31.3)
Dietitian	-			-	2 (12.5)
Psychologist	-			-	2 (12.5)
Physiotherapist	-			-	5 (31.3)
Occupational therapist	-			-	1 (6.2)
Speech and language therapist	-			-	1 (6.2)
Work experience, years	-			-	14.3 ± 8.9
Setting, *n* (%)					
Hospital	-			-	10 (62.5)
Primary care centre	-			-	6 (37.5)
Kinship with the person with CRD, *n* (%)					
Spouse			-	4 (80.0)	-
Son/Daughter			-	1 (20.0)	-
Duration of caregiving, *n* (%)					
<1 year			-	1 (20.0)	-
1–4 years			-	0 (0)	-
>4 years			-	4 (80.0)	-

Results are expressed as mean ± SD unless otherwise stated. AECOPD, acute exacerbations of COPD; BMI, body mass index; CAT, COPD assessment test; COPD, chronic obstructive pulmonary disease; CRD, chronic respiratory disease; FEV_1_, forced expiratory volume in 1 s; FVC, forced vital capacity; GOLD, global initiative for chronic obstructive lung disease; ILD, interstitial lung disease; LTOT, long-term oxygen therapy; NIV, non-invasive ventilation.

## Data Availability

The data presented in this study are available on request from the corresponding author.
